# The diagnostic performance of novel techniques for the detection of acute myocarditis: a clinical study using cardiovascular magnetic resonance imaging

**DOI:** 10.1186/1532-429X-15-S1-P162

**Published:** 2013-01-30

**Authors:** Vanessa Ferreira, Stefan K Piechnik, Erica Dall'Armellina, Theodoros Karamitsos, Jane M Francis, Ntobeko Ntusi, Cameron Holloway, Robin P Choudhury, Attila Kardos, Matthew D Robson, Matthias G Friedrich, Stefan Neubauer

**Affiliations:** 1 Cardiovascular Medicine, University of Oxford, Oxford, UK; 2 Cardiovascular Medicine, University of Calgary, Calgary, AB, Canada; 3 Cardiology, Milton Keynes NHS Hospital Foundation Trust, Milton Keynes, UK; 4 Cardiology, Université de Montréal, Montréal, QC, Canada

## Background

The accurate diagnosis of acute myocarditis on cardiovascular magnetic resonance imaging (CMR) often requires multiple modalities, including T2-weighted (T2W), early and late gadolinium imaging. Novel CMR techniques are now available, including bright-blood T2W-CMR, and T1-mapping which is also sensitive to changes in free water content. We hypothesized that these emerging methods can serve as new diagnostic criteria for myocarditis.

## Methods

**Figure 1:**
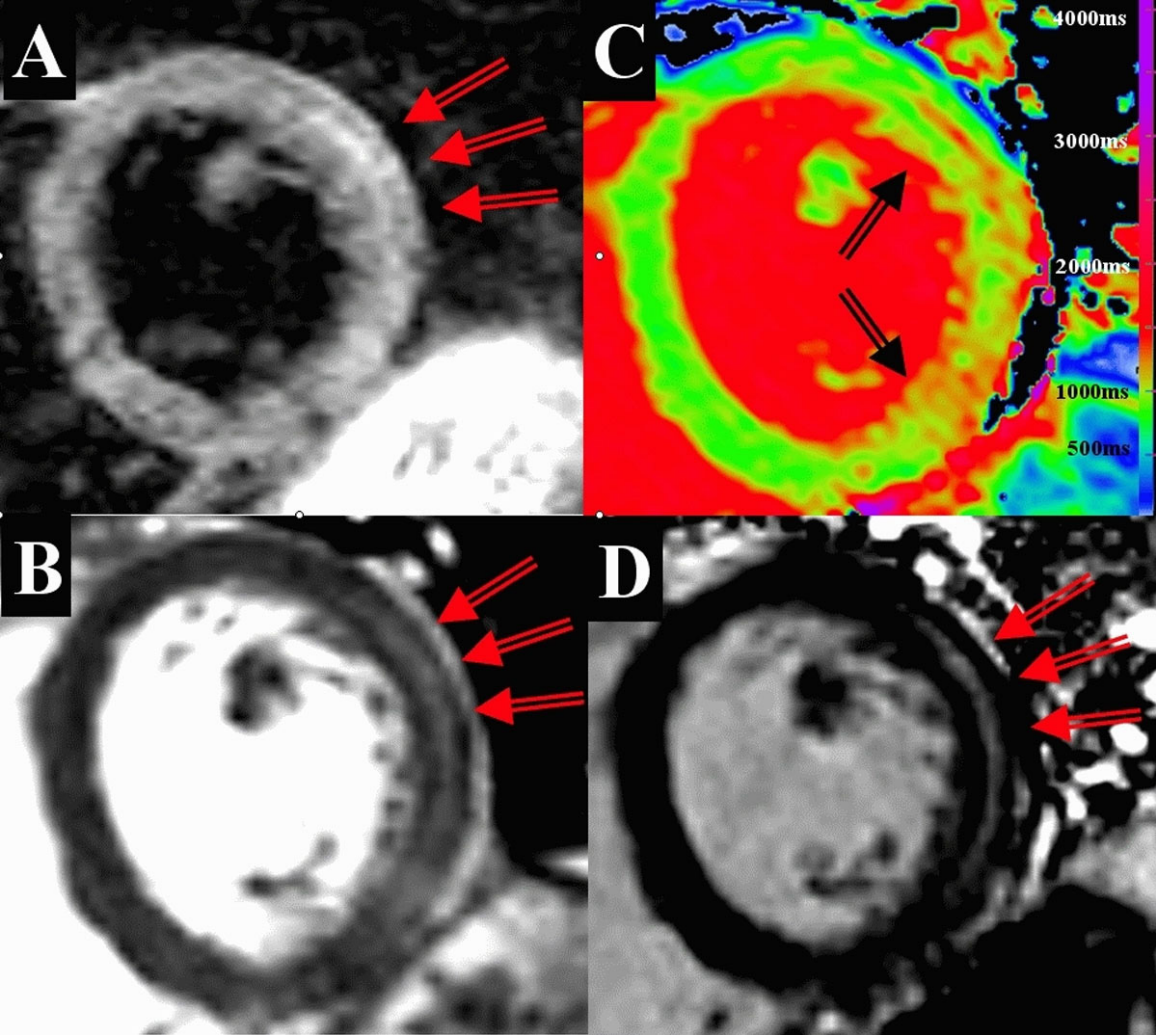
Acute myocarditis. (A) Dark-blood T2W-CMR showing increased signal intensity in the lateral wall. (B) Bright-blood T2W-CMR showing increased signal intensity in the mid lateral wall (C) T1-map showing increased T1 values (1100-1200 ms) in the lateral wall. (D) LGE imaging showing mid-wall enhancement in the lateral wall.

We studied 34 patients with suspected acute myocarditis and 45 healthy controls. All patients presented with chest pain, troponin I > 0.04 ug/L and had unobstructed coronary arteries on angiogram or ruled out clinically (e.g. young age < 35 years). CMR at 1.5T within 12 days of presentation included (1) dark-blood T2 (STIR); (2) bright-blood T2 (ACUT2E); (3) T1-mapping (ShMOLLI); and (4) late gadolinium enhancement (LGE) (Fig 1). Image analysis was performed for (1) global myocardial T2 signal intensity (SI) ratio against skeletal muscle; (2) mean myocardial T1; (3) LGE.

## Results

**Figure 2:**
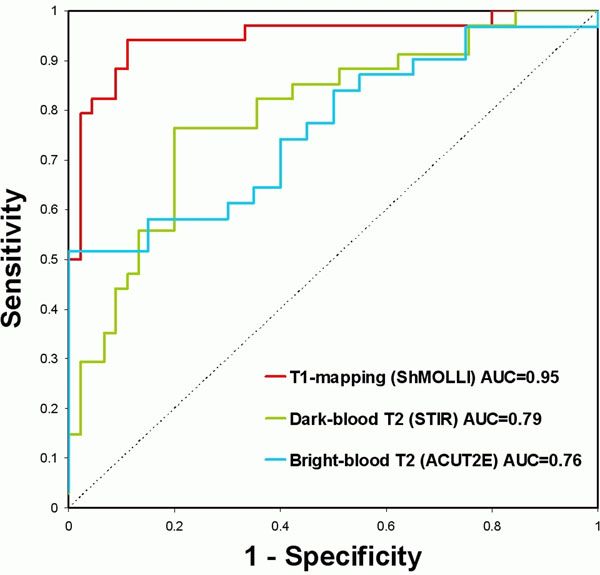
ROC curves for ShMOLLI T1-mapping, dark-blood T2W and bright-blood T2W-CMR in the detection of acute myocarditis.

All patients had a CMR diagnosis of acute myocarditis based on both positive T2-STIR and typical LGE pattern. Compared to controls, patients had significantly higher global myocardial T2 SI ratios by dark-blood T2W-CMR (1.81+/-0.28 vs 1.58+/-0.16, p<0.001), bright-blood T2W-CMR (2.90+/-0.33 vs 1.82+/-0.19, p<0.001) and mean myocardial T1 (1027+/-62 ms vs 942+/-21 ms, p<0.001). Receiver operator characteristic analysis showed good diagnostic performance for all methods, with T1-mapping having a significantly larger area-under-the-curve (0.95) compared to dark-blood T2W (0.79) and bright-blood T2W imaging (0.76; p<0.001 for both comparisons; Fig 2).

## Conclusions

T1-mapping showed superior diagnostic performance compared to conventional dark-blood and newer bright-blood T2W-CMR in the detection of acute myocarditis. T1-mapping and bright-blood T2W-CMR may be used as novel diagnostic criteria for the assessment of acute myocarditis.

## Funding

This study is funded by the Oxford National Institute for Health Research Biomedical Research Centre Programme. VMF is funded by the Alberta Innovates Health Solutions (AIHS) Clinical Fellowship and the University of Oxford Clarendon Fund Scholarship. Dr. Robin Choudhury is a Wellcome Trust Senior Research Fellow in Clinical Science. Stefan Neubauer and Robin Choudhury acknowledge support from the British Heart Foundation Centre of Research Excellence, Oxford

